# A commentary on the National Medical Licensing Examination in Vietnam: why, what, who and how

**DOI:** 10.12688/mep.19654.2

**Published:** 2023-09-06

**Authors:** Thuy Minh Ha

**Affiliations:** 1 College of Health Sciences, VinUniversity, Hanoi, Vietnam

**Keywords:** evaluation and assessment, national licensing exam, medical education, graduate outcome

## Abstract

As a result of increasing societal demands and economic development, the number of medical schools in Vietnam has increased significantly over the past decade. In order to ensure physician competency, it is imperative that medical training meets a minimal threshold before entering clinical practice. The prospects of the National Medical Licensing Exam (NMLE) have been discussed extensively and are expected to be instrumental in influencing curriculum reform, thus enhancing the quality of medical education. This paper discusses briefly why NMLE is necessary for Vietnam, what should be considered when establishing it, who could be the responsible organization, and how good practices can be learned and used as personal recommendations for educators and policymakers.

## Introduction

The number of medical schools in Vietnam has doubled since 1997 as a result of growing societal demands and economic development ( Word Health Organization, 2021). There are 29 medical schools in both public and private sectors, with an estimated more than eight thousand students graduating yearly ( Duong *et al*., 2021). There is concern about the competency of the newly graduated physicians due to the need for more rigorous quality assurance corridors. As of today, specific accreditation standards do not exist for medical training ( Ha & Siddiqui, 2022). Medical licensure exam is not yet required for medical graduates throughout the country. Medical practice licenses are currently based on the review of historical practice documents rather than an evaluation of physicians' competency. An applicant for a medical practice license must have a minimum of 18 months of practice as a physician, with a written certification of this period ( Government, 2022). Before 2024, the license is considered permanent, with no requirement for renewal ( Government, 2022). It implies that each medical school is responsible for the graduate outcome, resulting in the major concern about medical training quality. A traditional discipline-based approach with heavy theoretical lectures is the primary method of teaching in most medical schools ( Fan *et al*., 2012). The curriculum framework requirement for medical training was issued in 2012 and urged to be updated ( Ministry of Education and Training, 2012). Graduated competencies were endorsed for general practitioners in 2015, however, more data is needed regarding how they are incorporated into the curriculum ( Ministry of Health, 2015). A wide variety of medical curricula are available, as well as different graduate requirements pertaining to exams and graduate research theses. Over the past few years, the prospects for the National Medical Licensing Exam (NMLE) have been frequently discussed in a variety of medical education forums. An NMLE could play an important role in ensuring that physicians possess a minimum level of competency prior to entering clinical practice, thereby improving patient outcomes. Furthermore, it is expected to be a driver of curriculum reform, which will subsequently improve the medical education system. This paper discusses briefly why the introduction of NMLE is necessary for Vietnam, what should be considered during establishing process, who could be the responsible organization, and how good practices can be learned and used as personal recommendations for educators and policymakers.

### Why is it necessary to establish NMLE in Vietnam?

As one of the Association of Southeast Asian Nations (ASEAN) members, Vietnam signed a mutual recognition agreement (MRA) in 2009 that allowed healthcare professionals to practice in a host country if they had completed the required professional medical training and received the professional medical qualification in their homeland ( ASEAN, 2009). Members of this agreement were required to establish regulations for the migration of healthcare professionals. As a result, five out of 10 ASEAN countries have organized NMLEs, including Thailand, Philippines, Indonesia, Malaysia, and Laos ( Kittrakulrat *et al.*, 2014; Nur Hidayah *et al*., 2020; Sonoda *et al*., 2017). Experiences and practices gained from neighboring countries differed greatly. Several concerns about the NMLE system were identified including the targeted populations (foreign doctors *versus* domestic graduates); language variation (local language versus partial English); which poses challenges to the free flow of medical professionals to practice medicine in any of the region's countries ( Kittrakulrat *et al*., 2014). It is, however, believed to be the first step towards achieving comparable standards of patient care and medical education in ASEAN, in accordance with the World Health Organization (WHO) strategy for universal health coverage ( Nur Hidayah *et al*., 2020). The employability for highly skilled physicians across the region will be significantly improved if the MRA goal is achieved. Foreign doctors and overseas trained doctors will be able to enter the country more freely, resulting in an increased number of medical practitioners, thus improving the shortage of healthcare professionals in Vietnam.

For the NMLE to be established in Vietnam, there are a number of challenges. It is obvious that one of the most noteworthy issues in Vietnam could be the varying levels of readiness among different medical universities. A number of institutions are embracing a reformative shift towards competency-based medical education, while others still face substantial deficiencies, lacking the necessary literature, evidence, or motivation to adopt such a change ( Tran *et al*., 2022). Although there are numerous challenges associated with the NMLE, such as resource consumption, insufficient validity, and reliability, and a lack of direct connection between the NMLE and patient care, the benefits still outweigh the risks ( Nur Hidayah *et al*., 2020) ( Wang, 2022). According to recent experiences from neighboring countries, NMLEs have had positive impacts, such as motivating policy changes, revisiting curriculums and assessment practices, enhancing learning resources and facilities, and strengthening collaboration between educational networks in order to improve physician competence ( Nur Hidayah *et al*., 2020).

NMLE may have the disadvantage of creating a barrier for medical students and limiting the number of qualified physicians. On the other hand, when it comes to quality assurance, NMLE can be a tool that can be used to benchmark medical schools across countries and levels of potential across the region, thereby encouraging them to improve their curricula and continuously strive for quality improvement ( Swanson & Roberts, 2016).

### What should be considered for the establishment?

In the first instance, the strategic plan for the establishment of NMLE must comply with the national legal framework and educational system. The medical doctoral program in Vietnam is a six-year undergraduate training program overseen by the Ministry of Education and Training and the Ministry of Health ( Ha & Siddiqui, 2022). Several regulatory documents will be affected by the birth of NMLE, including but not limited to the Law on Medical Examination and Treatment, National Qualification Framework, Regulations on specialist training in the health sciences. Thus, a comprehensive strategic plan for the introduction of NMLE, which involves early involvement of the stakeholders, and an effective communication plan is essential to the project's success. A benchmarking with other countries' medical qualification systems is strongly recommended to facilitate a "fair exchange" of physician workforces within the ASEAN region ( Kittrakulrat *et al*., 2014).

Resistance to change is predictable in response to the NMLE establishment. Nevertheless, if the benefits of the institutions are emphasized, the top-down forces would be balanced with the institutions' benefits, which could increase achievement chance. As an example, low performing institution can be traced based on the NMLE results and offer more support for remediation, hence improving the educational outcomes ( Saleem & Afzal, 2022). As of 2015, the competency for general practitioners has been endorsed, which can serve as the very first starting point for the development of the NMLE by creating the blueprints ( Ministry of Health, 2015). Thus, it motivates schools to implement competency-based curricula, striving for higher quality in response to the upcoming NMLE. The time allocated for the exam should be considered if it will be an additional exam or if it will replace the existing tests from the schools, thus reducing workloads for faculty and students. Considering that most medical students are interested in specialist training ( Ngo, 2021), NMLE can also serve as an entry test for these courses after graduation.

The language used in the examination is also an important factor to consider. In seven out of 10 countries, English is being used for examinations in part or in full ( Kittrakulrat *et al*., 2014). The inclusion of English in this examination to some extent, similar to the Philippines and Malaysian models, could be an option for responding to the MRA. This notion finds further support in the ongoing admission trend in Vietnam, where the demand for English proficiency is increasingly recognized ( Nga, 2022). Nevertheless, the associated challenges merit careful consideration, ideally accompanied by a well-executed transition and preparation plan. These challenges encompass the potential language barrier that could hinder the understanding of complex medical concepts by both faculty and students ( Oducado *et al*., 2020). Additionally, it raises concerns about equity and accessibility, especially for students disadvantaged by limited exposure to English during their education ( Chur‐Hansen *et al*., 1997). Furthermore, there's a need to be mindful of cultural sensitivity; inaccurately communicated medical terms might lead to misinterpretations. This transition undoubtedly requires time to yield a clear understanding of its impact on healthcare quality ( Price *et al*., 2018). Ultimately, the primary objective of medical education is to foster capable doctors capable of delivering superior patient care. Should the emphasis shift excessively towards language proficiency at the expense of medical knowledge and skills, the quality of healthcare provided by graduates could be compromised. A one-size-fits-all approach is not possible when it comes to the NMLE. For an examination of this high stakes nature, it should also be considered whether: (i) it is only applicable to graduating medical students within the country to pass the exam before being granted a license to practice, or (ii) all prospective physicians from national jurisdictions or foreign medical graduates (IMGs) are required to pass the exam in order to practice within that jurisdiction ( Price *et al*., 2018). While the second option may require an additional process for this activity, NMLE represents a national minimum standard for evaluating doctors regardless of where they were trained.

### Who could be the responsible organization for Vietnam?

Generally, the National Medical Council, Medical Board, or Professional Associations under the Ministry of Health are responsible for organizing the NMLE (Professional Medical Regulatory) ( Kittrakulrat *et al*., 2014). In 2020, Vietnam National Medical Council (VNMC) was established in collaboration with WHO and the Vietnam Ministry of Health ( Prime Minister of the Government, 2020). According to the decision, the VNMC is responsible for defining, developing, implementing, monitoring, and assessing health professionals' competency. A national network of medical professionals (Vietnam Association for Medical Education – VAME) has been established in September 2022 under the guidance of the Vietnam Medical Council. These developments provide excellent momentum for NMLE development. In addition, several countries have recognized the importance of learning from this process and have created efforts to make the organization / professional body the foundation for accreditation agencies as well as be recognized by the World Federation of Medical Education Recognition Program ( Word Federation For Medical Education).

### How could it be learned from the current practices?

Medical graduates who wish to enter clinical practice could also be required to meet the NMLE as a minimum requirement. Registration renewal can also be considered every five years based on an evaluation of their participation in continuing professional development activities (CME/CPD). This contributes to the promotion of the continuing medical education (CME/CPD) system and ensures that health professionals are up to date on their knowledge and skills.

Several steps can be taken to prepare for high-stakes exams, as suggested by Roberts and colleagues: (i) blueprinting educational objectives and competency standards, (ii) selecting appropriate test formats, (iii) applying assessment strategies to ensure adequate reliability, and (iv) implementing appropriate standard-setting and decision-making procedures ( Roberts *et al*., 2006). A review of the Egyptian Medical License Exam Roadmap can also benefit for the purpose of NMLE establishment, as it consists of two main sections: *(i)* exam logistics and *(ii)* exam set up ( Nasser *et al.*, 2021). The logistics of the exam include the composition of the exam committee, the prerequisites for the exam, the admission criteria and fees, and the validity of the license. The exam set up encompasses the examination structure, the standard setting, the pass mark policy, and the reset policy ( Nasser *et al*., 2021). A multidisciplinary team was proposed for the exam committee, along with convenient infrastructure, logistics, and human resources. There are multiple articles that raise the same concern regarding exam transparency and accountability that must be maintained at every stage of the examination process ( Park & Yang, 2015) ( Sloane & Kelly, 2003). All stakeholders involved, from the exam committee to the administrative staff, should operate with the utmost transparency in their actions and decisions. Secondly, a robust system of checks and balances should be established ( Roberts *et al.*, 2006). This involves maintaining separation between various functions within the examination process to prevent undue influence or manipulation. Those responsible for setting exam standards should not select questions. There should be mechanisms in place to cross-verify and validate decisions made at different stages. Furthermore, diversifying the exam committee composition remains essential. Including professionals from a variety of disciplines and backgrounds can minimize bias and foster a more comprehensive perspective ( Thompson, 2006). Rigorous training and clear codes of conduct should be enforced for committee members to ensure they uphold fairness and objectivity.

Rolling out a new high-stakes exam requires careful planning. The timeline for recognizing new results and dealing with pre-exam graduates should be clear ( Saleem & Afzal, 2022). Communication strategies should inform both schools and students. Continuous improvement is key through collaboration, adjustments, and refining content based on feedback ( Bajammal *et al.*, 2008). In conclusion, meticulous planning and transparent communication are essential for a successful transition to a new high-stakes exam, benefiting medical education and healthcare quality.

## Conclusion

The establishment of the National Medical Licensing Exam in Vietnam may present numerous challenges, but it is expected to positively impact curriculum changes and enhance the mobility of the medical workforce in the region. The introduction of an NMLE is a time-critical issue that requires the attention of all stakeholders within the network. Several aspects of the licensing system remain to be considered and further research is required. By publishing this paper, the author hopes to contribute to discussion among regulators and policymakers in order to reach a consensus for the best interests of the country. 

## Data Availability

No data are associated with this article.
