# Supplementary material for: A commentary on the National Medical Licensing Examination in Vietnam: why, what, who and how
Source: MedEdPublish (2016). 2023 Sep 6;13:30. Originally published 2023 May 24. [Version 2] doi: 10.12688/mep.19654.2 (PMC10518846; doi:10.12688/mep.19654.2)
Supplement: Peer review history

# Reviewer report: Reviewer response for version 2

**DOI:** 10.21956/mep.21192.r34768

**Published:** 2023-09-20

**Authors:** Todd M. Pollack, David Duong, Le Ngoc Bao

**Affiliations:** 1 Department of Medicine, Beth Israel Deaconess Medical Center, Boston, MA, USA; 2 Department of Global Health and Social Medicine, Harvard Medical School, Cambridge, MA, USA; 3 The Partnership for Health Advancement in Vietnam, Beth Israel Deaconess Medical Center, Hanoi, Vietnam

Thanks to the authors for thoroughly addressing our comments.  We have just one small comment/edit:  

 In the paragraph that begins "The language used in the examination is also an important factor to consider. In seven out of 10 countries....."  Please clarify your meaning. Does it mean 7 out of 10 countries 'in the ASEAN region', for example?

Is the topic of the opinion article discussed accurately in the context of the current literature?

Yes

Are arguments sufficiently supported by evidence from the published literature?

Yes

Are all factual statements correct and adequately supported by citations?

Yes

Are the conclusions drawn balanced and justified on the basis of the presented arguments?

Yes

Reviewer Expertise:

Medical education

We confirm that we have read this submission and believe that we have an appropriate level of expertise to confirm that it is of an acceptable scientific standard.

# Reviewer report: Reviewer response for version 2

**DOI:** 10.21956/mep.21192.r34767

**Published:** 2023-09-20

**Authors:** Gail Morrison

**Affiliations:** 1 The Perelman School of Medicine, University of Pennsylvania, Philadelphia, Pennsylvania, USA

Additional paragraphs have added substantial data and information for the implementation of a NLME in Vietnam .

Is the topic of the opinion article discussed accurately in the context of the current literature?

Yes

Are arguments sufficiently supported by evidence from the published literature?

Yes

Are all factual statements correct and adequately supported by citations?

Yes

Are the conclusions drawn balanced and justified on the basis of the presented arguments?

Yes

Reviewer Expertise:

NA

I confirm that I have read this submission and believe that I have an appropriate level of expertise to confirm that it is of an acceptable scientific standard.

# Reviewer report: Reviewer response for version 1

**DOI:** 10.21956/mep.21054.r33577

**Published:** 2023-07-19

**Authors:** Todd M. Pollack, David Duong, Le Ngoc Bao

**Affiliations:** 1 Department of Medicine, Beth Israel Deaconess Medical Center, Boston, MA, USA; 2 Department of Global Health and Social Medicine, Harvard Medical School, Cambridge, MA, USA; 3 The Partnership for Health Advancement in Vietnam, Beth Israel Deaconess Medical Center, Hanoi, Vietnam

This opinion article presents issues surrounding the establishment of the National Medical Licensing Examination (NMLE) in Vietnam. The author discusses why an NMLE is necessary, how it should be established, and reviews relevant literature to identify good practices and lessons learned. The topic is relevant and timely as the establishment of the National Medical Council (NMC) and the NMLE in Vietnam are areas of current active discussion. As the author points out, recent developments in policy and legislation, including the revision of the Law on Examination and Treatment, and the recent establishment of the NMC have created positive conditions for the establishment of the NMLE. Importantly, the National Medical Council is mandated to set and regulate medical standards and to guide the organization and implementation of the NMLE. Additionally, the author discusses some arguments in favor of establishing the NMLE, mentions anticipated challenges, and reviews good practices and lessons learned from other settings; particularly from the Egyptian experience. Overall, the article raises some important considerations and relevant issues, supported by the current literature, and applied to the Vietnam context. 

 The reviewers have the following comments for the author’s consideration

1. The article would benefit from a more detailed discussion of the anticipated challenges to the establishment and implementation of the NMLE in Vietnam. Barriers such as resource consumption, insufficient validity and reliability of exams, and a lack of direct relation between NMLE and patient care are appropriately mentioned. However, additional challenges in the Vietnam setting could also include the challenges related to the development of the exam (should it include multiple-choice-questions only or also include a skills examination component?) and the differences in the level of readiness among different medical universities. For the latter, it may be worth going into more detail on the current ongoing process of medical curricular reform toward competency-based medical education (see: Tran TD, Vu PM, Pham HTM, Au LN, et al.: Transforming medical education to strengthen the health professional training in Viet Nam: A case study. *Lancet Reg Health West Pac*. 2022; **27**: 100543 ^1^)
2. The author discusses the importance of including the medical school network in the process of developing the NMLE. The development and creation of an NMLE process and the development of the NMLE needs to be transparent with buy-in from all stakeholders involved, including patients. The purpose of an NMLE needs to be clearly defined and agreed upon. If, as the author indicates, the purpose is for standardization and to help promote health care quality, then consideration of those on the receiving end of health care (i.e. patients) needs to be included.
3. The article would benefit from mentioning, aside from the need for an NMLE, the implementation of the exam as well - how to ensure that it is fair, free from corruption, and from competing interests. 
4. Additional consideration of the roll-out/phase-in of such an exam needs to be mentioned - when will the results of the exam be in full effect, what will happen to those physicians who graduated prior to the exam's roll out, how does the NMC prepare the schools for the exam?
5. The authors mention the benefits of using English language for the NMLE. Is that the recommendation for Vietnam at the current time?  What are the challenges?
6. The lessons learned section draws heavily on the Egyptian experience. Are there other county experiences that might be worth including as well?

Is the topic of the opinion article discussed accurately in the context of the current literature?

Yes

Are arguments sufficiently supported by evidence from the published literature?

Yes

Are all factual statements correct and adequately supported by citations?

Yes

Are the conclusions drawn balanced and justified on the basis of the presented arguments?

Yes

Reviewer Expertise:

Medical education

We confirm that we have read this submission and believe that we have an appropriate level of expertise to confirm that it is of an acceptable scientific standard, however we have significant reservations, as outlined above.

## References

[1] : Transforming medical education to strengthen the health professional training in Viet Nam: A case study. *Lancet Reg Health West Pac* .2022;27: 10.1016/j.lanwpc.2022.100543 100543 10.1016/j.lanwpc.2022.100543 35874914PMC9301568

# Reviewer report: Reviewer response for version 1

**DOI:** 10.21956/mep.21054.r33578

**Published:** 2023-07-13

**Authors:** Gail Morrison

**Affiliations:** 1 The Perelman School of Medicine, University of Pennsylvania, Philadelphia, Pennsylvania, USA

A very well written and organized commentary on implementing a National Medical Licensing Exam (NMLE) in Vietnam. The commentary proposes that passing such a required exam for all medical school graduates would assure a minimum level of competency entering clinical practice, be a driver of curriculum reform, and most likely result in improving patient outcomes. 

 The commentary is thoughtfully organized into four sections. The first section: Why is it necessary to establish a NMLE. This section emphasizes that Vietnam is a member of the Association of Southeast Asian Nations (ASEAN) and 5 of the 10 countries have implemented a NMLE. Vietnam has also signed a Mutual Recognition Agreement (MRA) in 2009 as well with these countries. Though there are both positive and negative advantages, the most significant advantage is that NMLE can be the tool used to benchmark medical schools across Vietnam.

 The second section: What should be considered for the establishment. Implementing such an exam most certainly will require compliance with the national legal framework and educational system of Vietnam. Benchmarking with other countries’ medical qualification system is strongly recommended. What language should exam be in, and who besides new graduates should take it. The Third section: Who could be the responsible organization for Vietnam. In 2020,Vietnam National Medical Council (VNMC) was established in collaboration with WHO and the Vietnam Ministry of Health and is responsible for defining, developing, implementing, monitoring and assessing health professionals’ competency. A national network of medical professional (Vietnam Association for Medical Education- VAME) has also been established. The fourth section: How could it be learned from current practices. A multidisciplinary team was proposed for the exam committee along with convenient infrastructure, logistics and Human Resources as initial steps to developing this high stakes exam, the NMLE utilizing published blueprints from other countries. In conclusion, the author hopes this commentary will contribute to discussions among regulators and policymakers regarding the introduction of an NMLE in Vietnam.

 Several comments for the author:

 In the Introduction, in sentence: There is a concern about the competency……., the sentence ends with rigorous quality assurance “corridors “? I have never seen “corridors “ used with quality assurance. 

 In section: What should Ministry of Education be Ministry of Education and Training (MOËT)?

 Opinion article comments: 

 The topic of the opinion article is discussed accurately in the context of the current literature

  All factual statements seem correct and adequately support by citations 

 ​​​​​​ The author’s statements and conclusions are sufficiently supported by evidence from the cited published literature. 

 The author’s conclusions are balanced and justified on the basis of the presented arguments 

 Overall, the author has written a well thought out commentary on the justification for a NMLE for Vietnam.

Is the topic of the opinion article discussed accurately in the context of the current literature?

Yes

Are arguments sufficiently supported by evidence from the published literature?

Yes

Are all factual statements correct and adequately supported by citations?

Yes

Are the conclusions drawn balanced and justified on the basis of the presented arguments?

Yes

Reviewer Expertise:

NA

I confirm that I have read this submission and believe that I have an appropriate level of expertise to confirm that it is of an acceptable scientific standard.
